# Supplementary material for: A new clustering and nomenclature for beta turns derived from high-resolution protein structures
Source: PLoS Comput Biol. 2019 Mar 7;15(3):e1006844. doi: 10.1371/journal.pcbi.1006844 (PMC6424458; doi:10.1371/journal.pcbi.1006844)
Supplement: S1 Supporting information — (PDF) [file pcbi.1006844.s001.pdf]

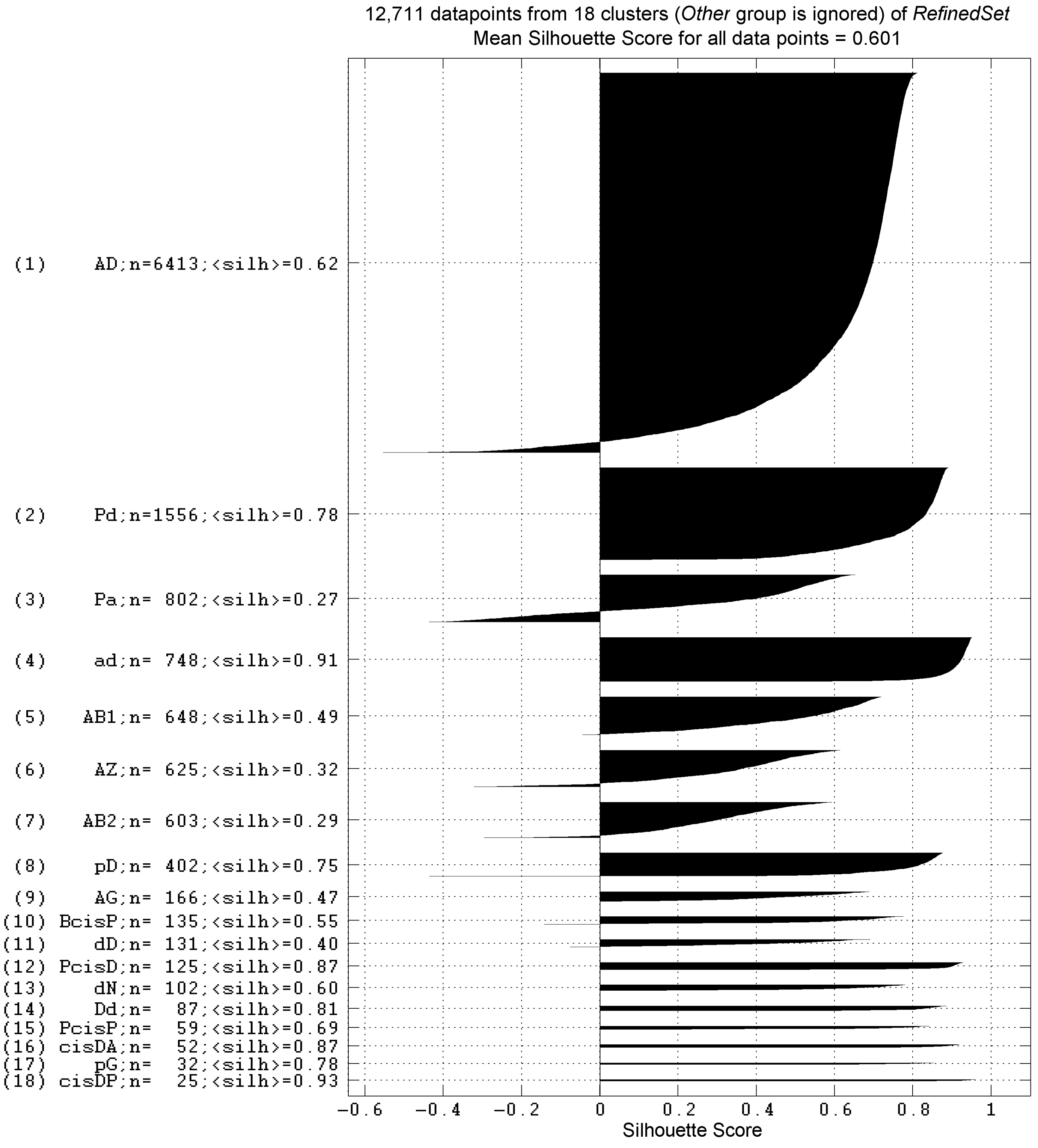

Fig A. Silhouette scores for all 18 clusters. The overall mean score is 0.601. Some of the clusters produced with k-medoids have lower scores in the (0.3, 0.7) range. DBSCAN failed to produce them in the first place because of their varying density, overlapping and spread-out nature. Some of the clusters have few individual data points with negative (-1.0, 0.0) scores, which occur in some of the subclusters generated by k-medoids. The silhouette score uses a different metric (average distance of a point to the members of a cluster) than k-medoids does (distance of a point to the medoid of a cluster), so that some of the k-medoids clusters have some negative silhouette scores. The silhouette score analysis provides an additional validation of the 18 produced clusters, also supported by distinct peaks in the density generated with a maximum-likelihood optimization and 10-fold cross validation.

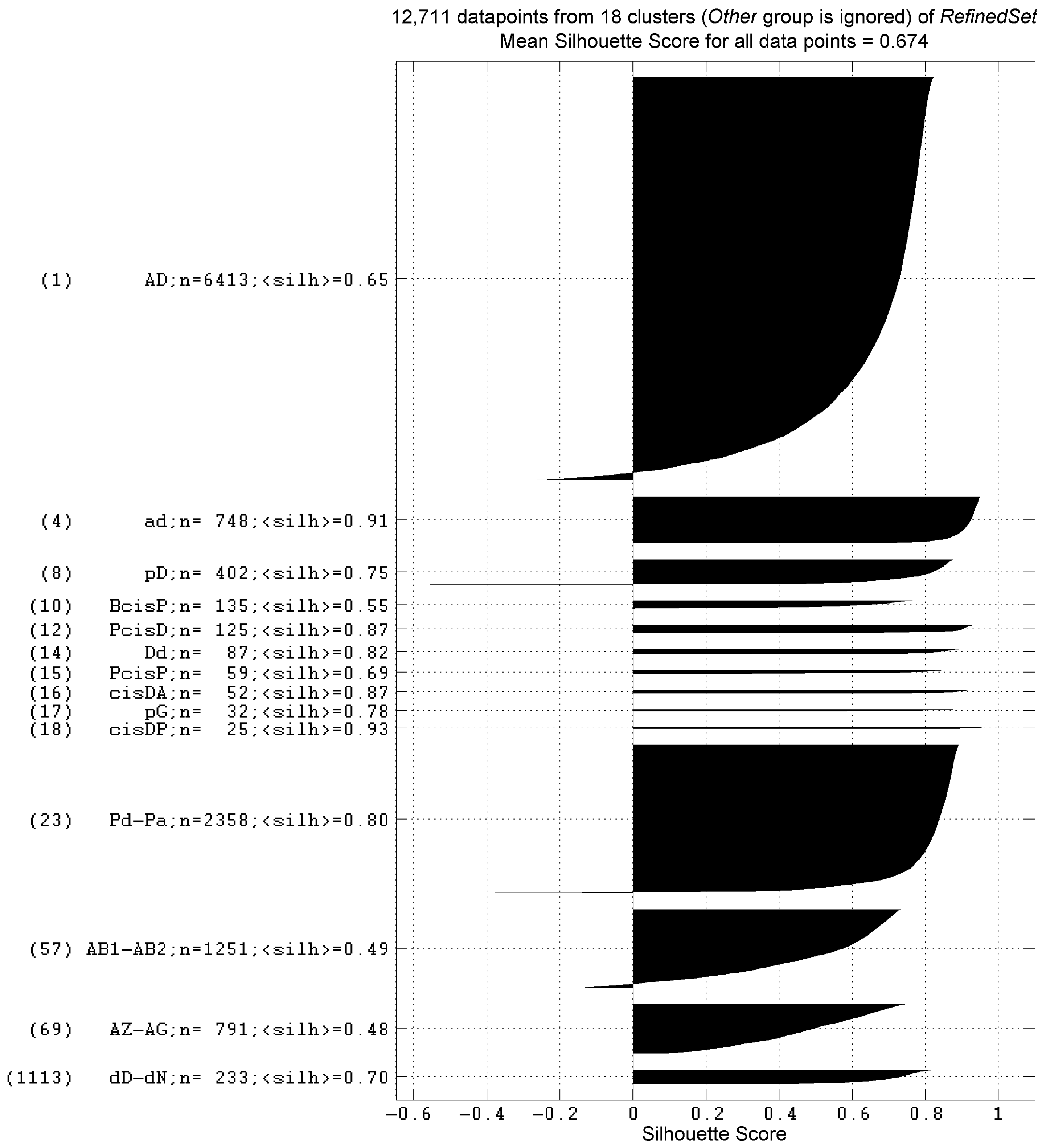

Fig B. Silhouette scores for 14 clusters after merging subclusters AB1 with AB2, AZ with AG, Pd with Pa, and dD with dN. The mean score of the points after merging is 0.674. The merged subclusters have better silhouette scores than the unmerged subclusters (Suppl. Fig A) but still represent peaks in the density in dihedral angle space and distinct sequence profiles.

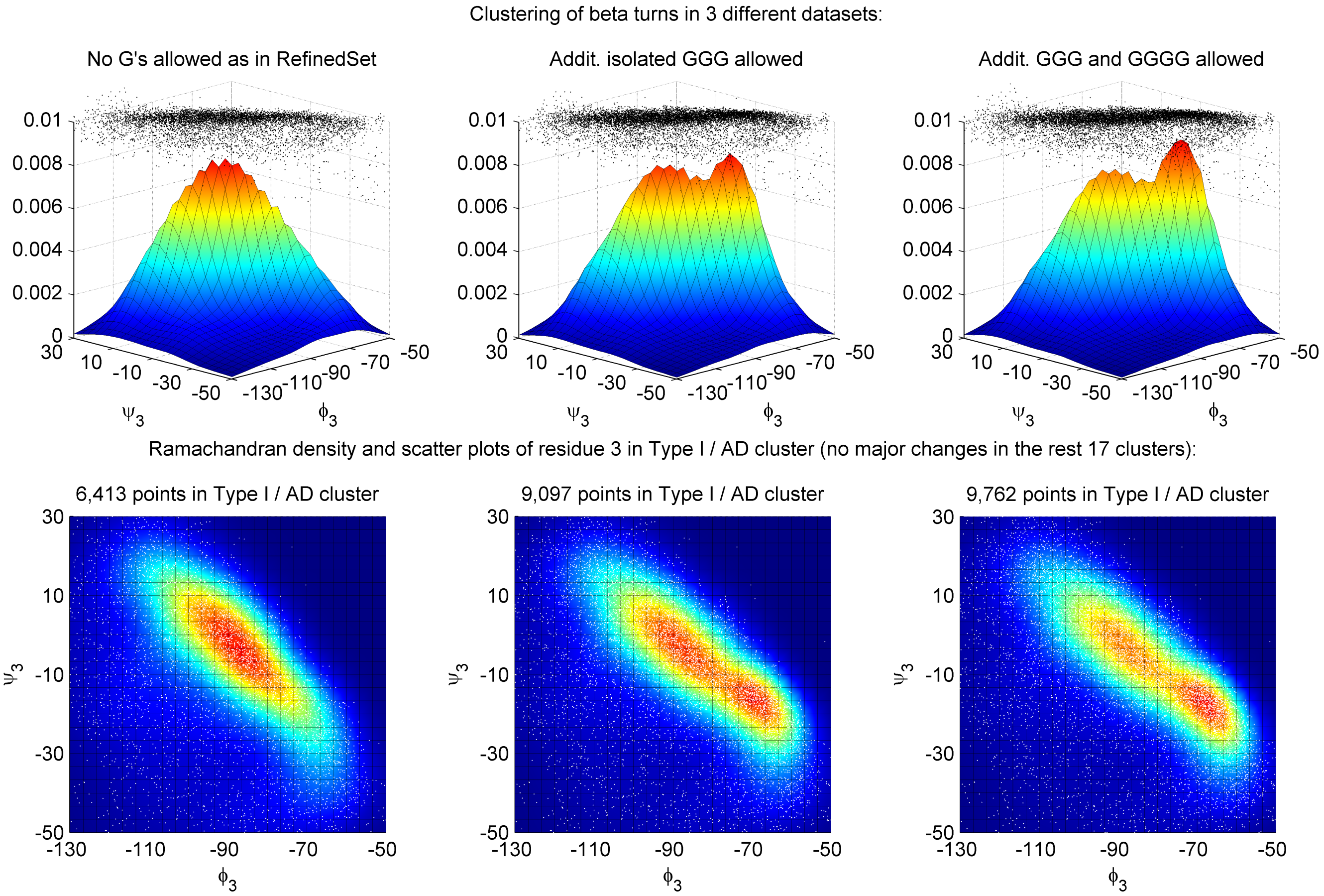

Fig C. Clustering results for the Type I / AD cluster for 3 cases from left to right: the original *RefinedSet* with no 3_{10} helix (DSSP code G) allowed; the *RefinedSet* with isolated 3-residue 3_{10} helices (GGG) added; and *RefinedSet* with both isolated 3- and 4-residue 3_{10} helices (GGG and GGGG) added. Isolated 3_{10} helices are those not immediately abutting alpha helices. The remaining 17 clusters do not have major changes. The number of beta turns increased by 42% and 52% for the AD cluster overall for GGG and GGG/GGGG respectively relative to no G's. The second peak near ϕ_3,ψ_3 = (-70°,-20°) becomes more prominent: 36% and 38% AA sub-population for GGG and GGG/GGGG vs. 28% for no G's. The formation of the additional cluster AA was rejected because it did not have significantly different aminoacid profile (Fig D in S1 Supporting Information) and the ϕ_3,ψ_3 conformation varies along the natural main diagonal.

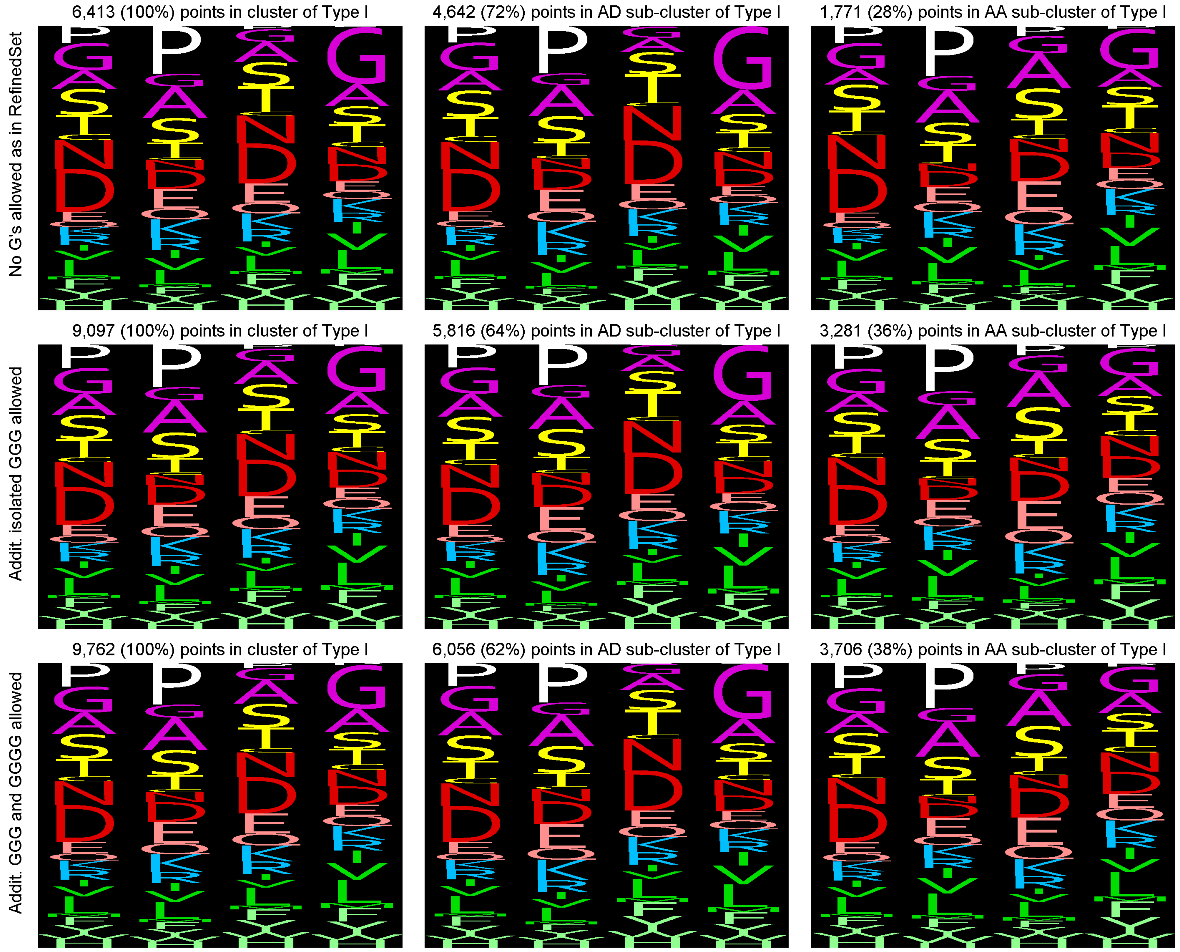

Fig D. Sequence profiles for clustering with and without 3_{10} helices. First row: *RefinedSet* with no G's; second row *RefinedSet* with isolated GGG segments added; third row, *RefinedSet* with GGG and GGGG added. First column, the AD+AA cluster; second column, the AD cluster; third column, the AA cluster. The AD / AA sub-clusters populations change from 72% / 28% (no G's) to 64% / 36% (+GGG) and 62% / 38% (+GGG and GGGG). The formation of the additional cluster AA was rejected because it did not have significantly different aminoacid profile.

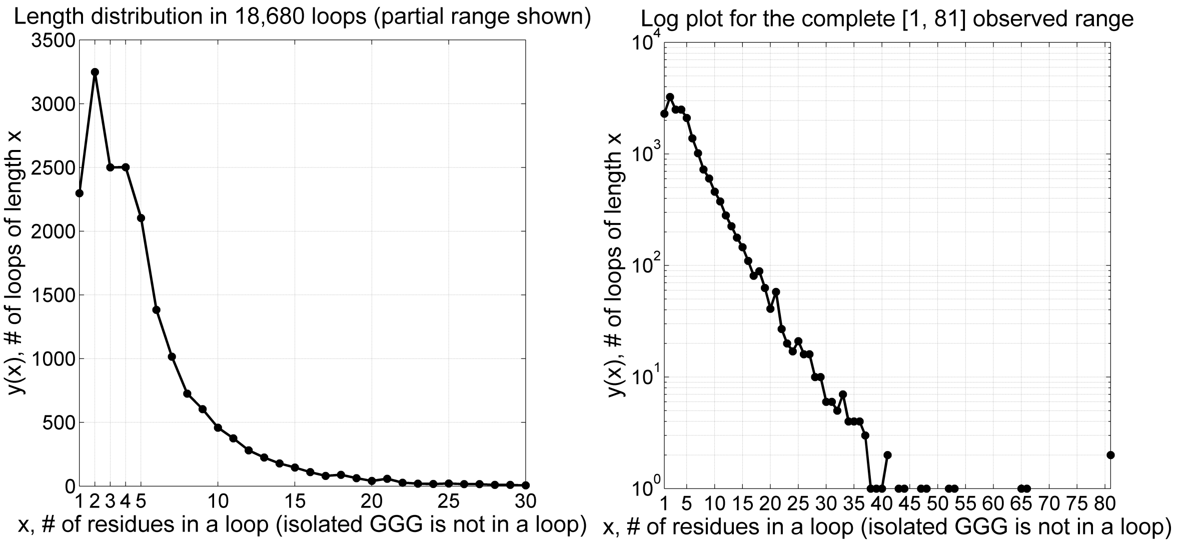

Fig E. Frequency of loop length in *CompleteSet* of 18,680 loops with all 3_{10} helices excluded from loops. The exclusion of GGG in the loop definition increases the total number of loops by 9% from 17,176 to 18,680, because some loops are split by 3-residue 3_{10} helices. The frequency of a loop still decreases exponentially after the length of 5 with approximately a 10-fold reduction every time a loop adds 10 more residues. 95% of loops are 1 to 13 residues long. Very long loops are rare, only 0.29% of loops are 30 or more residues long.

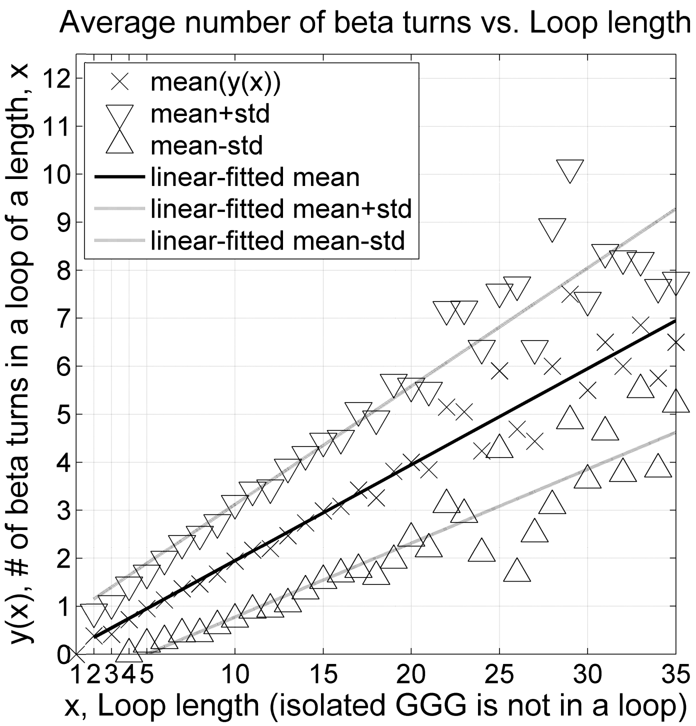

Fig F. Average number of beta turns in loops as a function of loop length when all 3_{10} helices are not considered part of loops. For every 5.5 loop residues, there is on average a single beta turn.
