# Supplementary material for: A new clustering and nomenclature for beta turns derived from high-resolution protein structures
Source: PLoS Comput Biol. 2019 Mar 7;15(3):e1006844. doi: 10.1371/journal.pcbi.1006844 (PMC6424458; doi:10.1371/journal.pcbi.1006844)
Supplement: S2 Supporting information — (PDF) [file pcbi.1006844.s002.pdf]

S2 Supporting Information. This file contains Ramachandran plots for all 18 clusters and *Other* turns when no Cα1-Cα4 distance cutoff is used. Points in blue have Cα1-Cα4 distance ≤ 7.0 Å; points in red have Cα1-Cα4 > 7 Å. The first column shows all points that can be assigned to the cluster by the dihedral angle metric regardless of Cα1-Cα4 distance. The second column shows data for those points with Cα1-Cα4 ≤ 7.0 Å. The third column shows data for those points with Cα1-Cα4 > 7 Å. The histograms in the bottom row show the distribution of Cα1-Cα4 distances for each group. There is one page for each cluster and for the *Other* group. Four-residue segments with Cα1-Cα4 up to about 8 Å are shaped like turns. Those above 7 Å are wider and shallower than classical beta turns (Cα1-Cα4 ≤ 7.0 Å). Segments with Cα1-Cα4 distance around 9 Å are L-shaped. Those at about 10 Å are significantly extended and similar to segments of beta sheet strands. Most 4-residue segments in the *Other* category are not shaped like turns since their Cα1-Cα4 distance is above 9 Å.

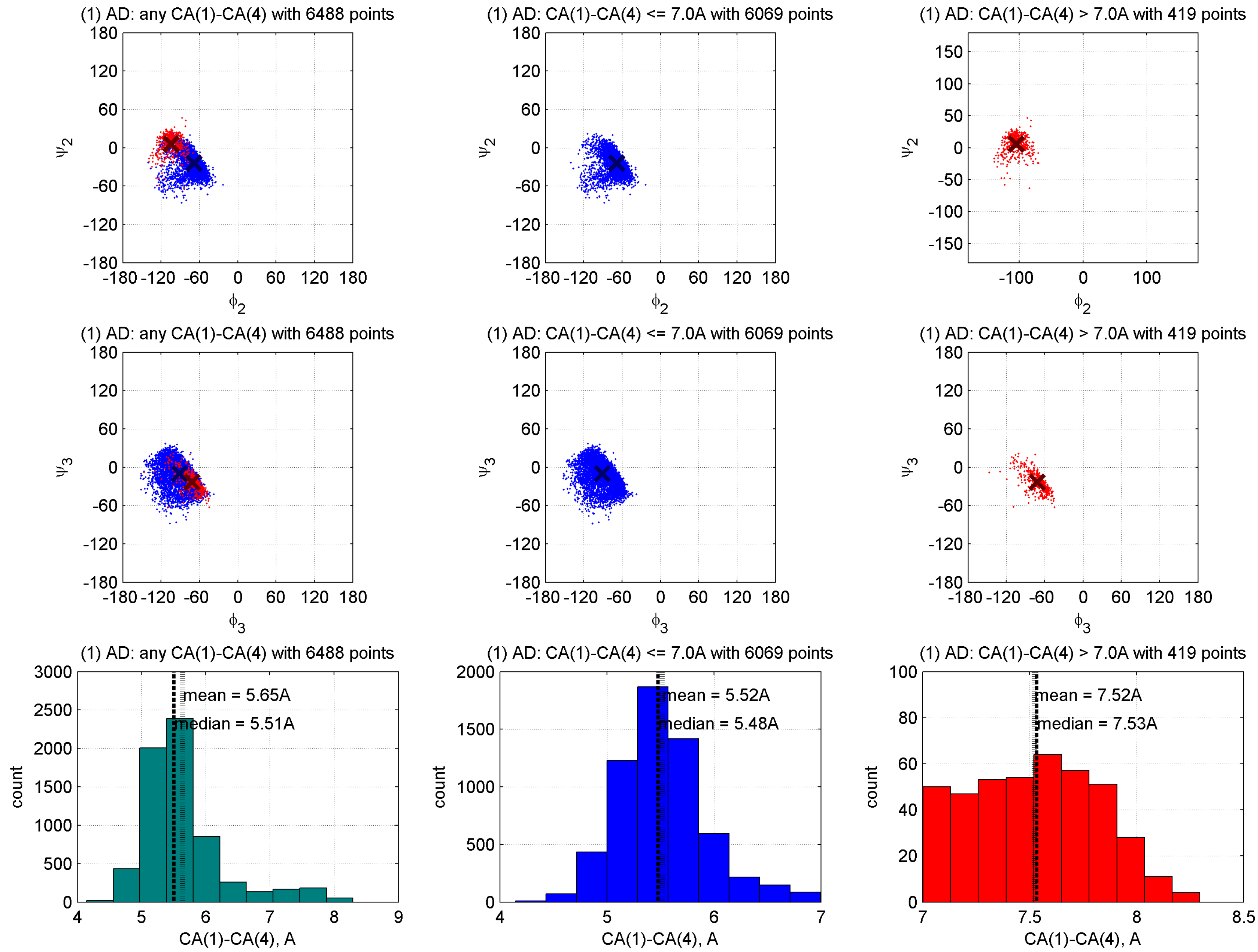

(1) AD: any CA(1)-CA(4) with 6488 points
(1) AD: CA(1)-CA(4) <= 7.0A with 6069 points
(1) AD: CA(1)-CA(4) > 7.0A with 419 points
ψ_2
ϕ_2
ψ_3
ϕ_3
mean = 5.65A
median = 5.51A
mean = 5.52A
median = 5.48A
mean = 7.52A
median = 7.53A
count
CA(1)-CA(4), A

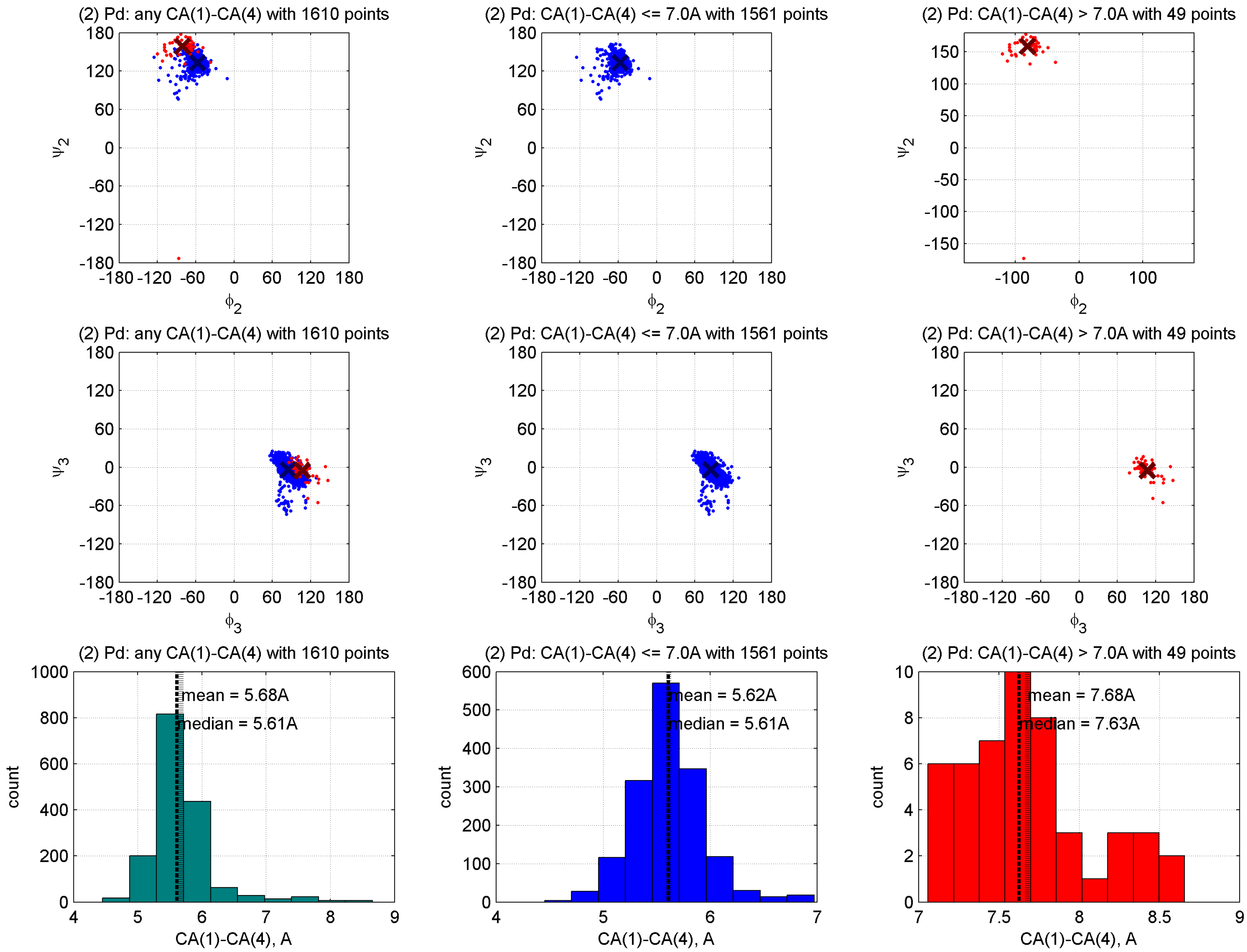

(2) Pd: any CA(1)-CA(4) with 1610 points
(2) Pd: CA(1)-CA(4) <= 7.0A with 1561 points
(2) Pd: CA(1)-CA(4) > 7.0A with 49 points
ψ2
φ2
ψ3
φ3
count
CA(1)-CA(4), A
mean = 5.68A
median = 5.61A
mean = 5.62A
median = 5.61A
mean = 7.68A
median = 7.63A

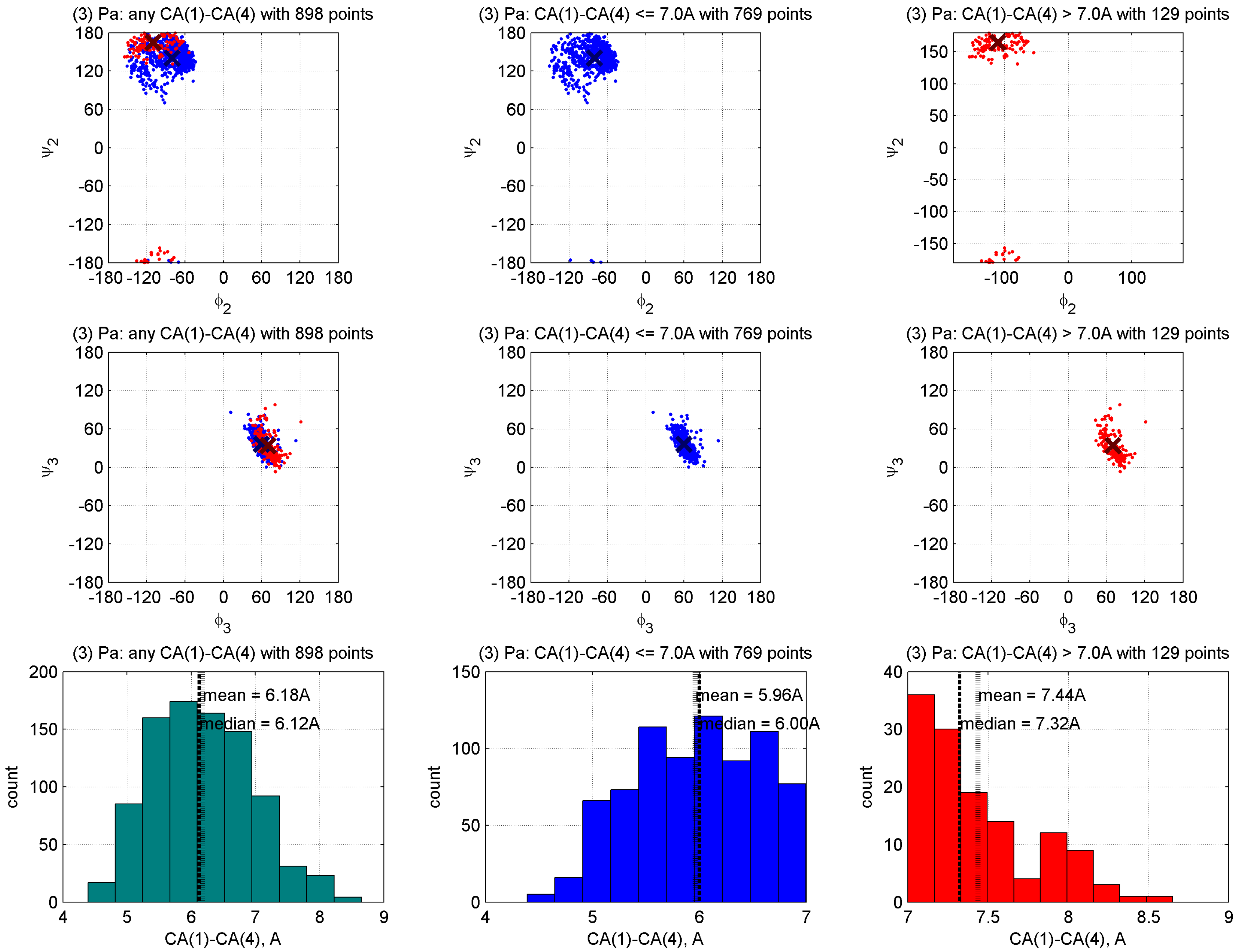

(3) Pa: any CA(1)-CA(4) with 898 points
(3) Pa: CA(1)-CA(4) <= 7.0A with 769 points
(3) Pa: CA(1)-CA(4) > 7.0A with 129 points
ψ_2
ϕ_2
ψ_3
ϕ_3
count
CA(1)-CA(4), A
mean = 6.18A
median = 6.12A
mean = 5.96A
median = 6.00A
mean = 7.44A
median = 7.32A

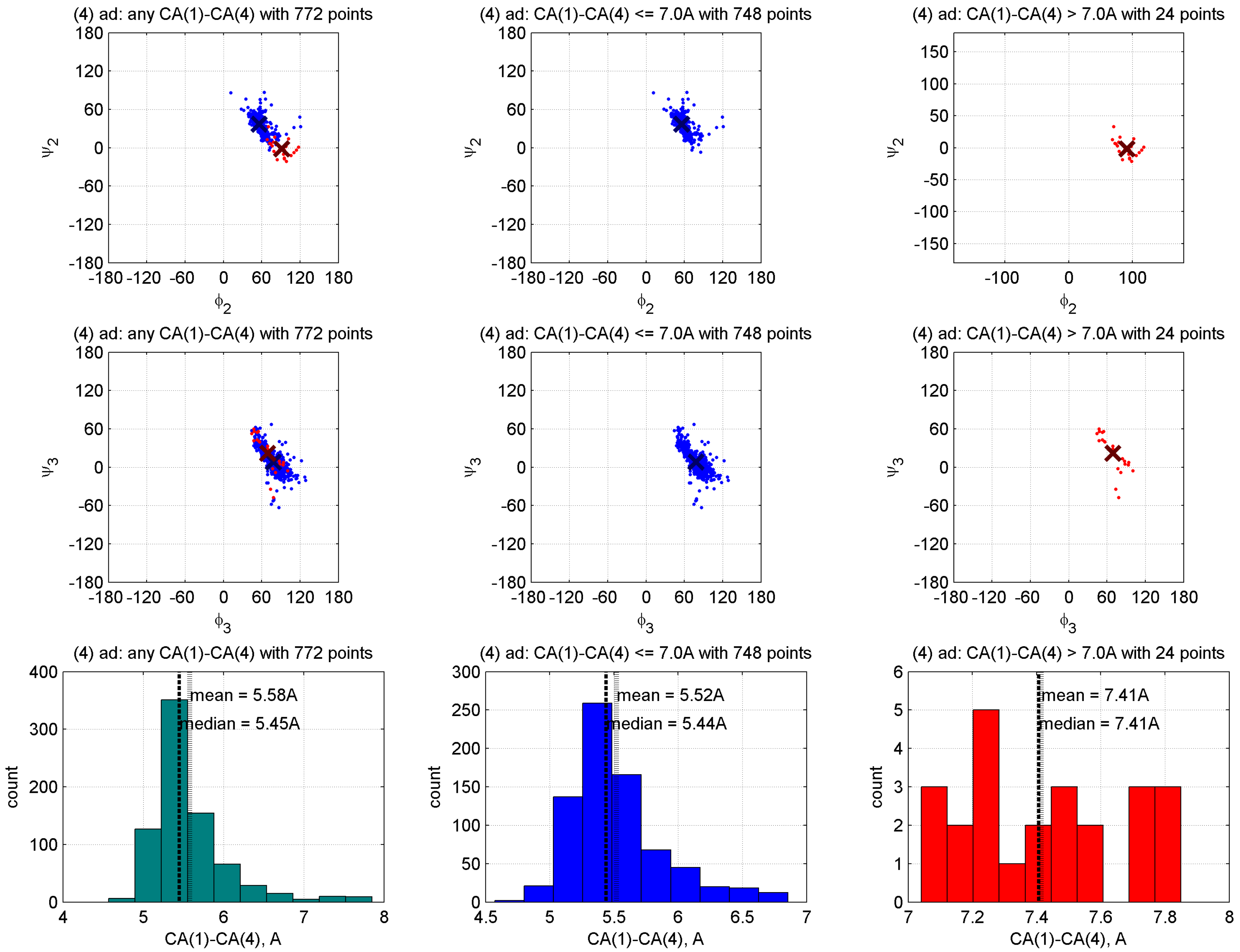
(4) ad: any CA(1)-CA(4) with 772 points
(4) ad: CA(1)-CA(4) <= 7.0A with 748 points
(4) ad: CA(1)-CA(4) > 7.0A with 24 points
ψ2
φ2
-180 -120 -60 0 60 120 180
-150 -100 -50 0 50 100 150
(4) ad: any CA(1)-CA(4) with 772 points
(4) ad: CA(1)-CA(4) <= 7.0A with 748 points
(4) ad: CA(1)-CA(4) > 7.0A with 24 points
ψ3
φ3
(4) ad: any CA(1)-CA(4) with 772 points
(4) ad: CA(1)-CA(4) <= 7.0A with 748 points
(4) ad: CA(1)-CA(4) > 7.0A with 24 points
count
CA(1)-CA(4), A
mean = 5.58A
median = 5.45A
mean = 5.52A
median = 5.44A
mean = 7.41A
median = 7.41A

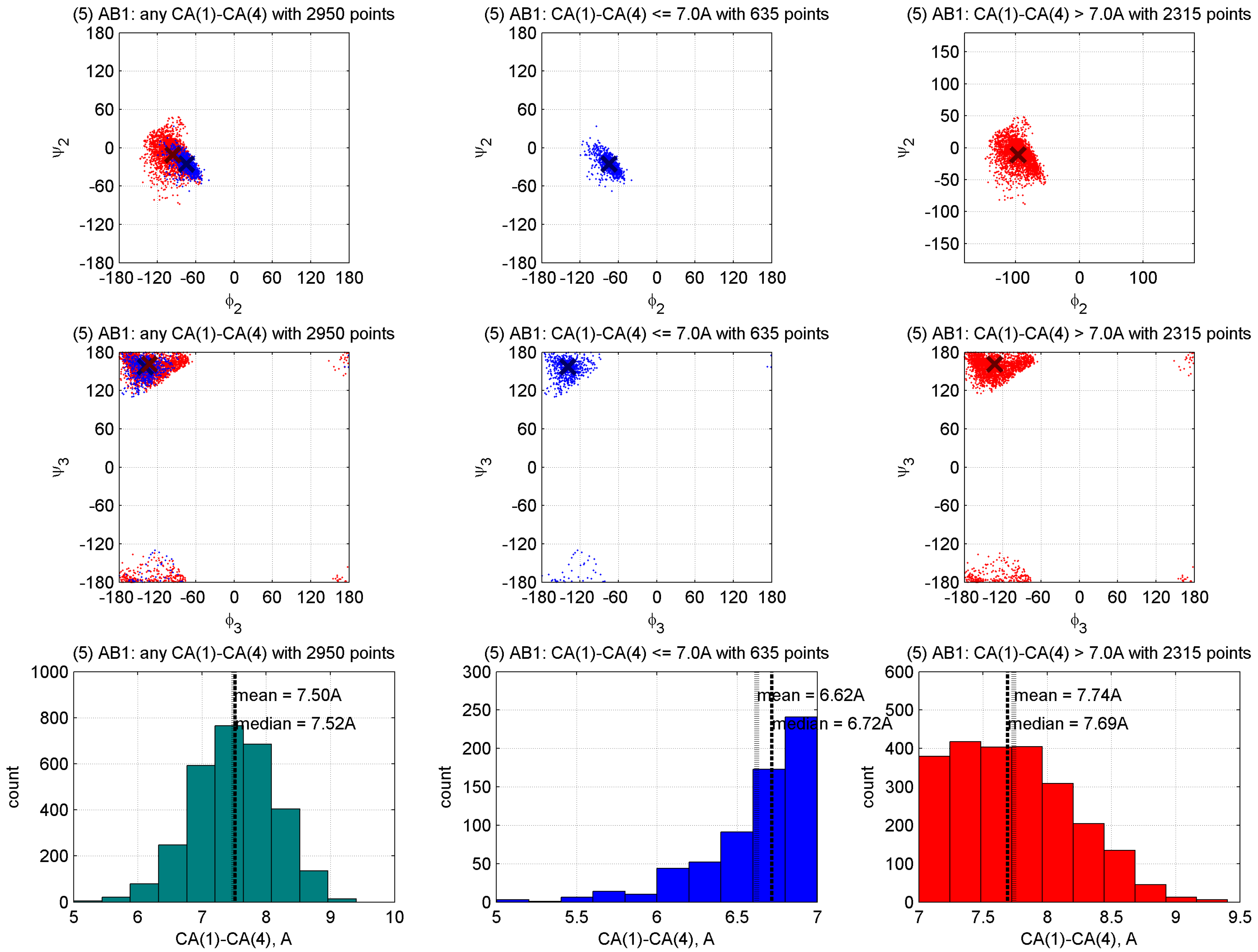
(5) AB1: any CA(1)-CA(4) with 2950 points
(5) AB1: CA(1)-CA(4) <= 7.0A with 635 points
(5) AB1: CA(1)-CA(4) > 7.0A with 2315 points
ψ_2
ϕ_2
ψ_3
ϕ_3
count
CA(1)-CA(4), A
mean = 7.50A
median = 7.52A
mean = 6.62A
median = 6.72A
mean = 7.74A
median = 7.69A

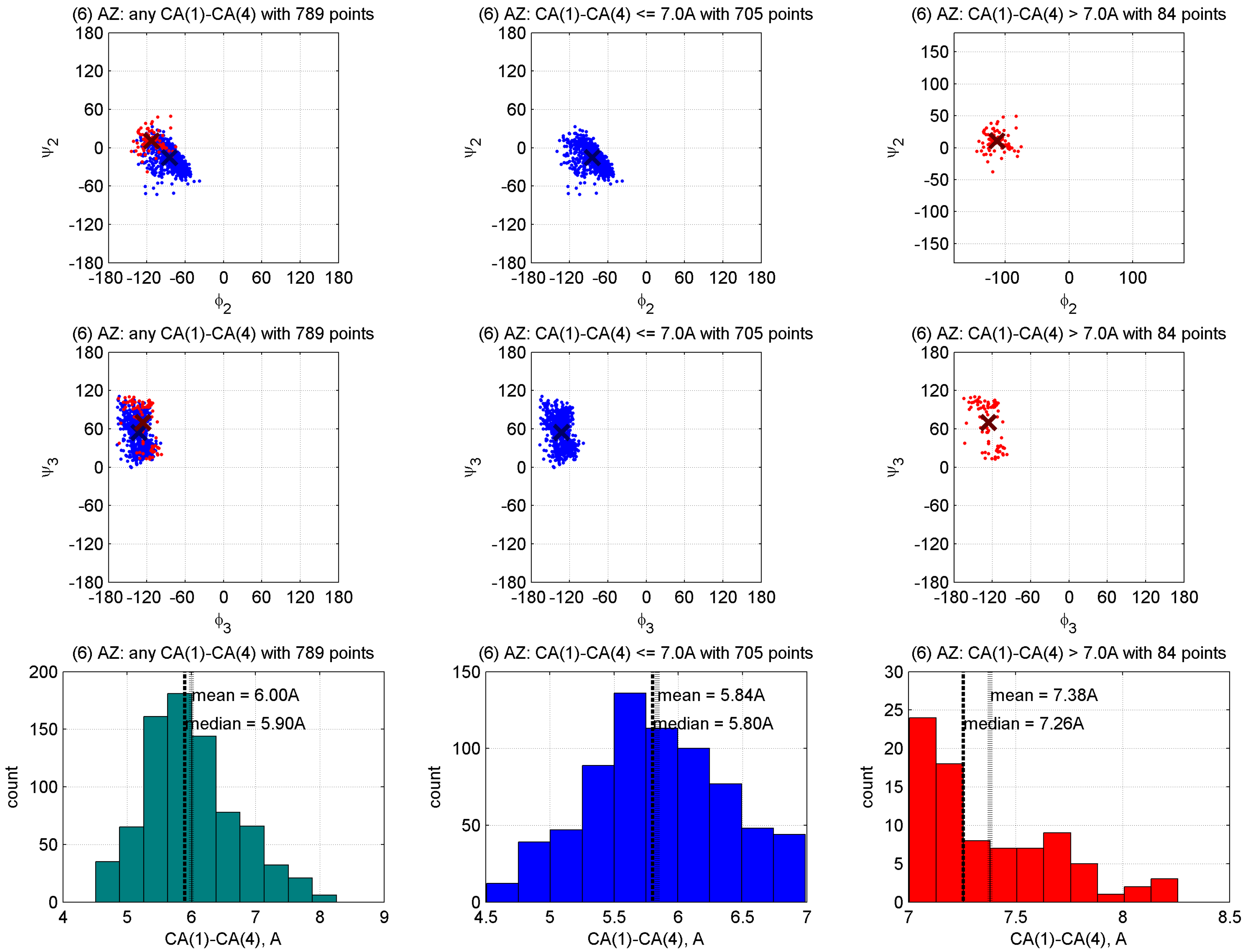
(6) AZ: any CA(1)-CA(4) with 789 points
(6) AZ: CA(1)-CA(4) <= 7.0A with 705 points
(6) AZ: CA(1)-CA(4) > 7.0A with 84 points
ψ2
φ2
ψ3
φ3
count
CA(1)-CA(4), A
mean = 6.00A
median = 5.90A
mean = 5.84A
median = 5.80A
mean = 7.38A
median = 7.26A

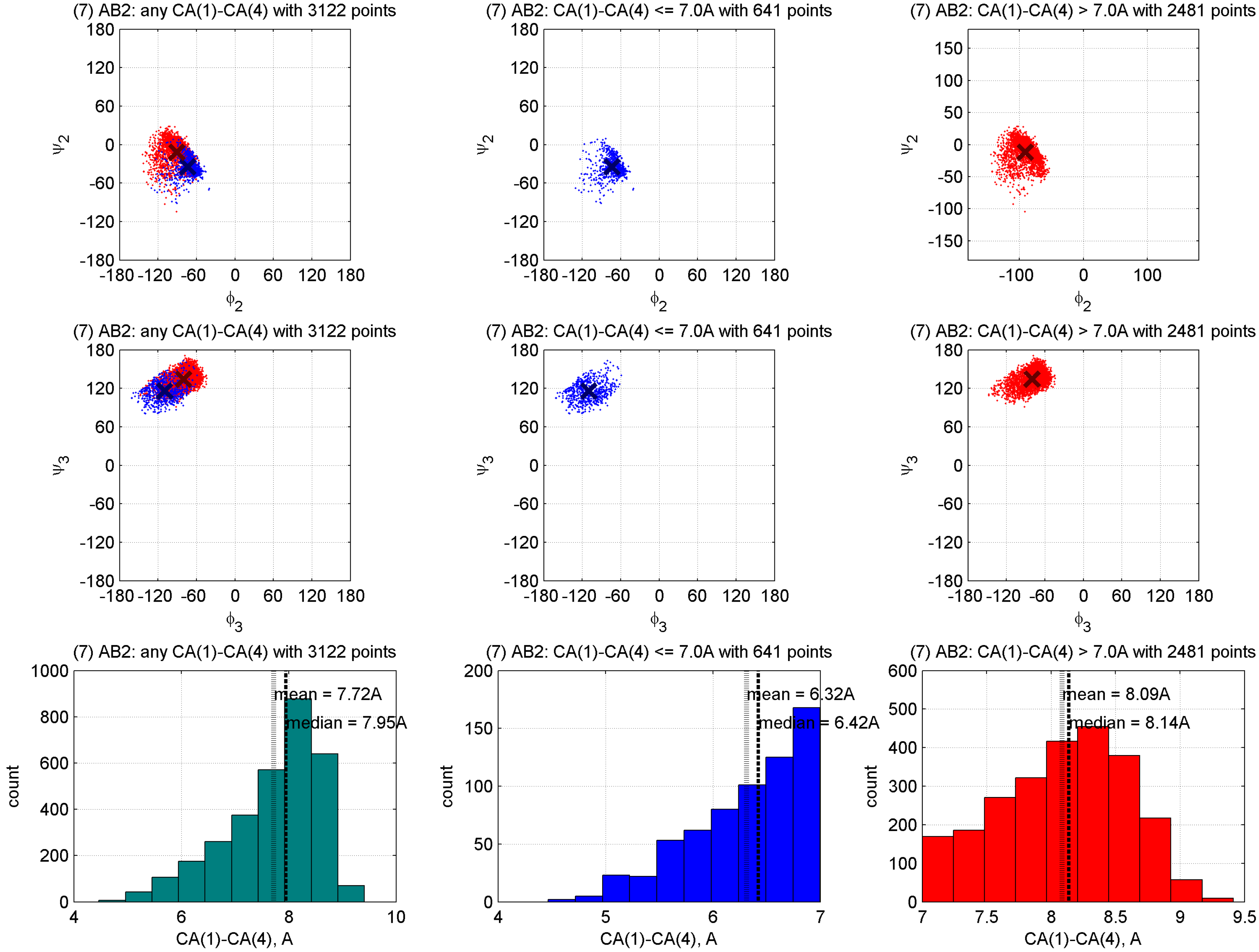

(7) AB2: any CA(1)-CA(4) with 3122 points
(7) AB2: CA(1)-CA(4) <= 7.0A with 641 points
(7) AB2: CA(1)-CA(4) > 7.0A with 2481 points
ψ_2
ϕ_2
ψ_3
ϕ_3
count
CA(1)-CA(4), A
mean = 7.72A
median = 7.95A
mean = 6.32A
median = 6.42A
mean = 8.09A
median = 8.14A

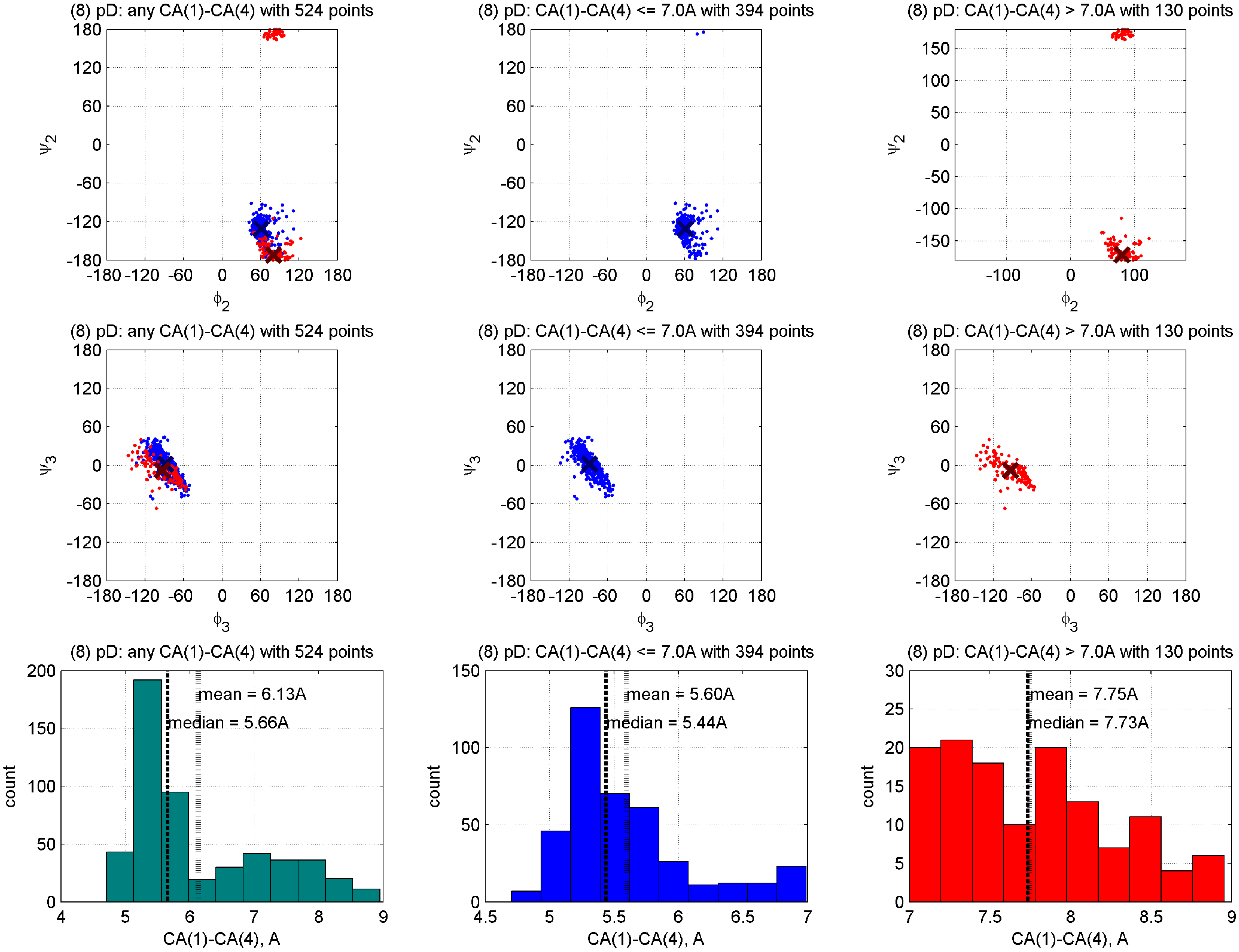

(8) pD: any CA(1)-CA(4) with 524 points
(8) pD: CA(1)-CA(4) <= 7.0A with 394 points
(8) pD: CA(1)-CA(4) > 7.0A with 130 points
ψ_2
ϕ_2
ψ_3
ϕ_3
count
CA(1)-CA(4), A
mean = 6.13A
median = 5.66A
mean = 5.60A
median = 5.44A
mean = 7.75A
median = 7.73A

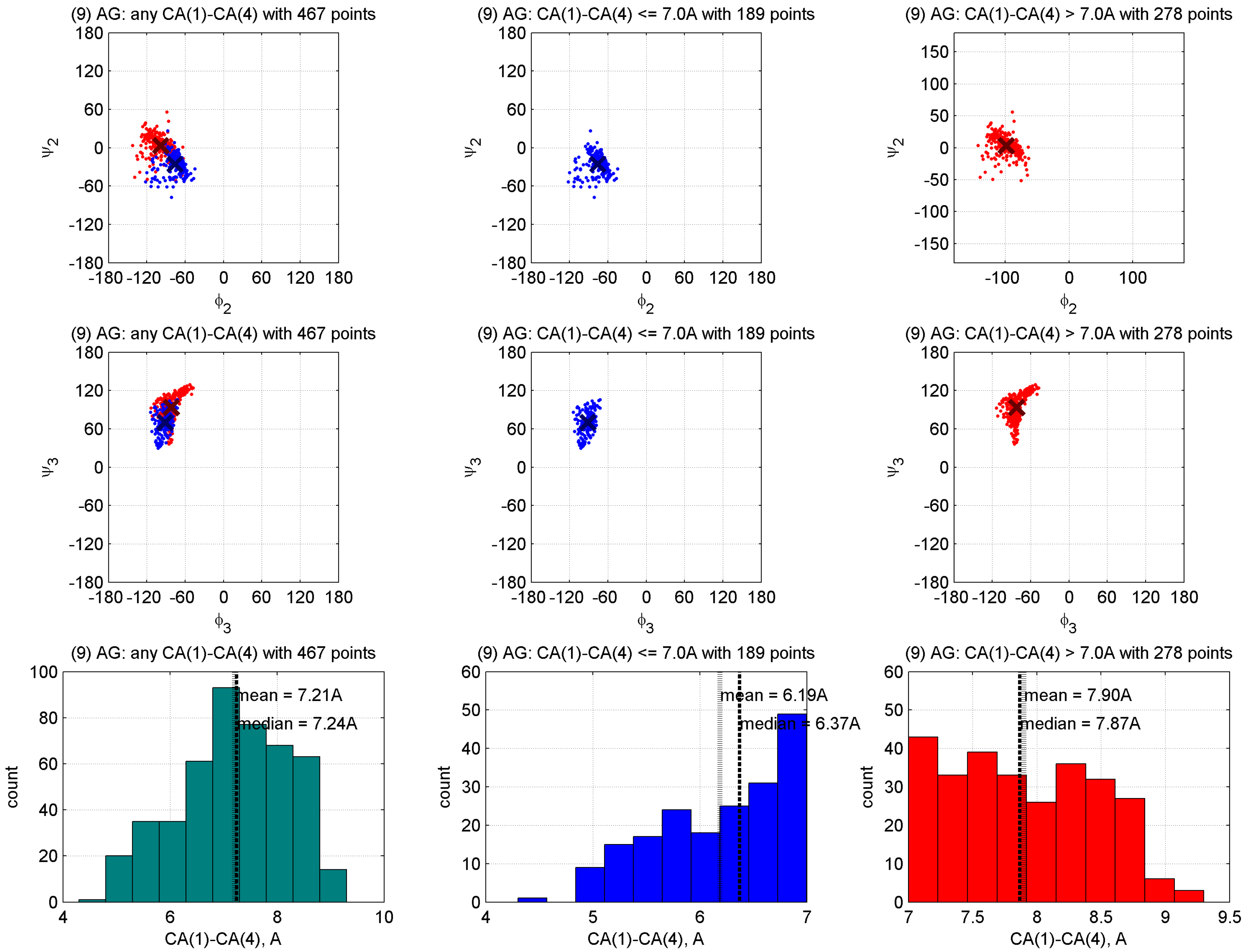

(9) AG: any CA(1)-CA(4) with 467 points
(9) AG: CA(1)-CA(4) <= 7.0A with 189 points
(9) AG: CA(1)-CA(4) > 7.0A with 278 points
ψ2
φ2
ψ3
φ3
count
CA(1)-CA(4), A
mean = 7.21A
median = 7.24A
mean = 6.19A
median = 6.37A
mean = 7.90A
median = 7.87A

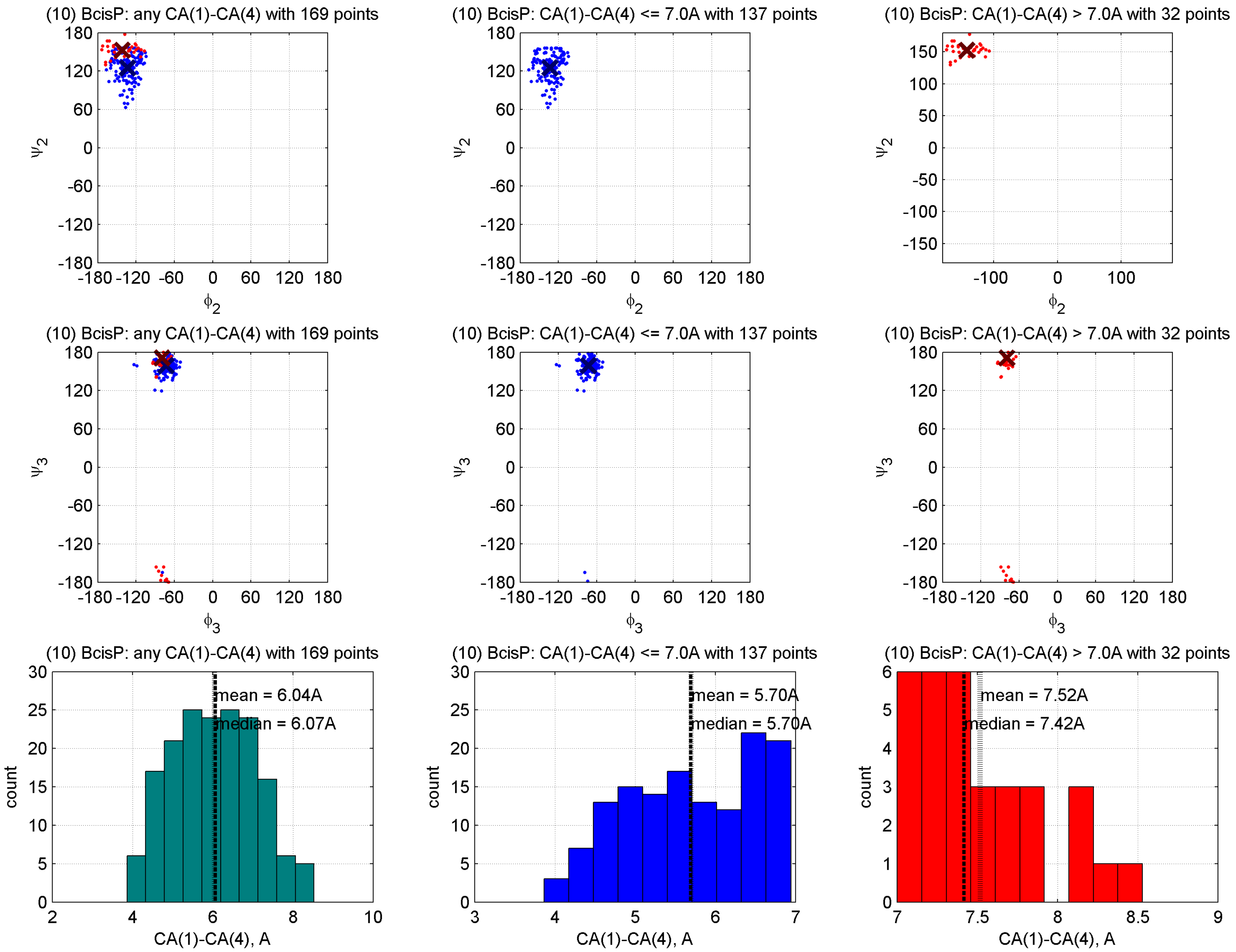
(10) BcisP: any CA(1)-CA(4) with 169 points
(10) BcisP: CA(1)-CA(4) <= 7.0A with 137 points
(10) BcisP: CA(1)-CA(4) > 7.0A with 32 points
ψ_2
ϕ_2
ψ_3
ϕ_3
count
CA(1)-CA(4), A
mean = 6.04A
median = 6.07A
mean = 5.70A
median = 5.70A
mean = 7.52A
median = 7.42A

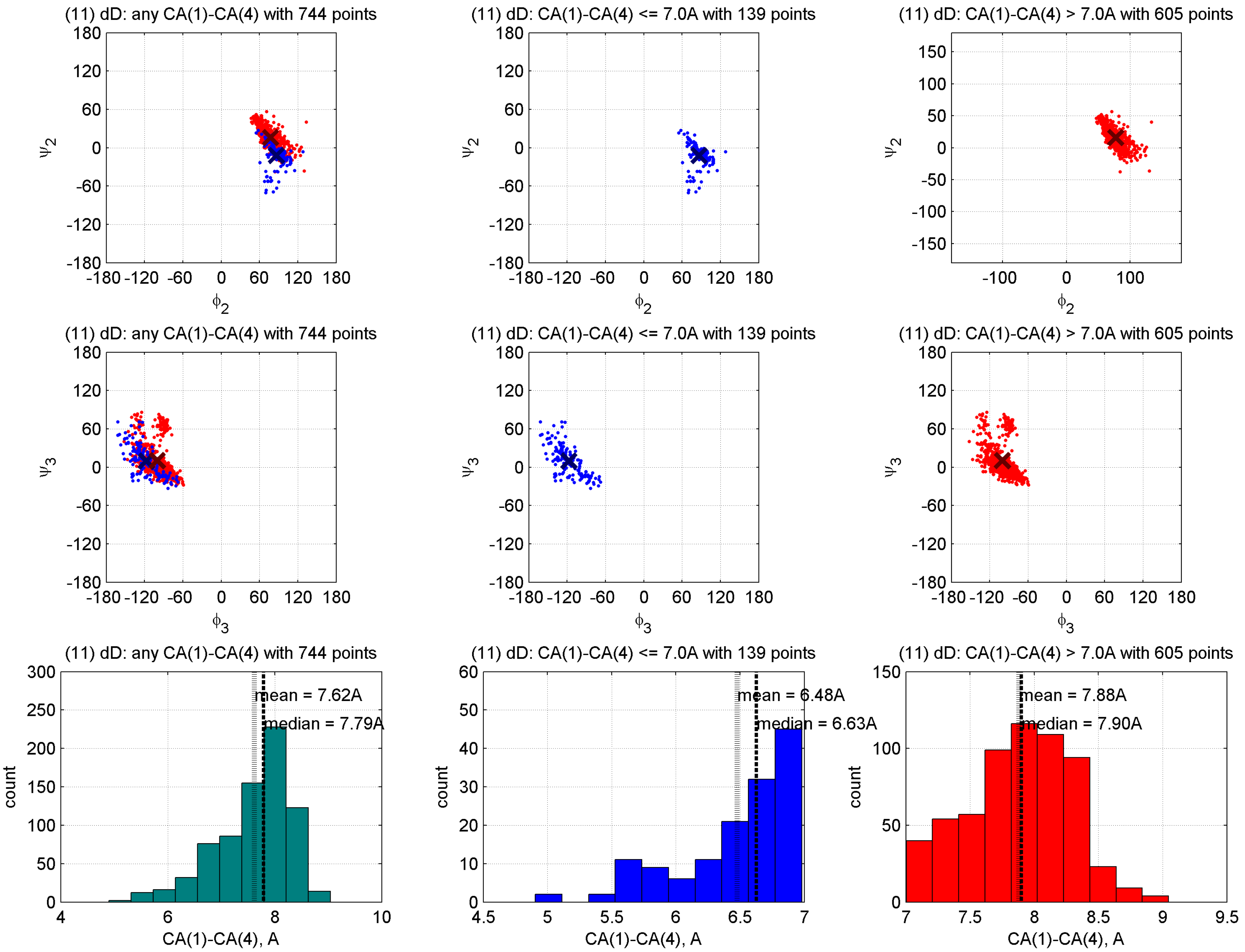
(11) dD: any CA(1)-CA(4) with 744 points
(11) dD: CA(1)-CA(4) <= 7.0A with 139 points
(11) dD: CA(1)-CA(4) > 7.0A with 605 points
mean = 7.62A
median = 7.79A
mean = 6.48A
median = 6.63A
mean = 7.88A
median = 7.90A
count
CA(1)-CA(4), A

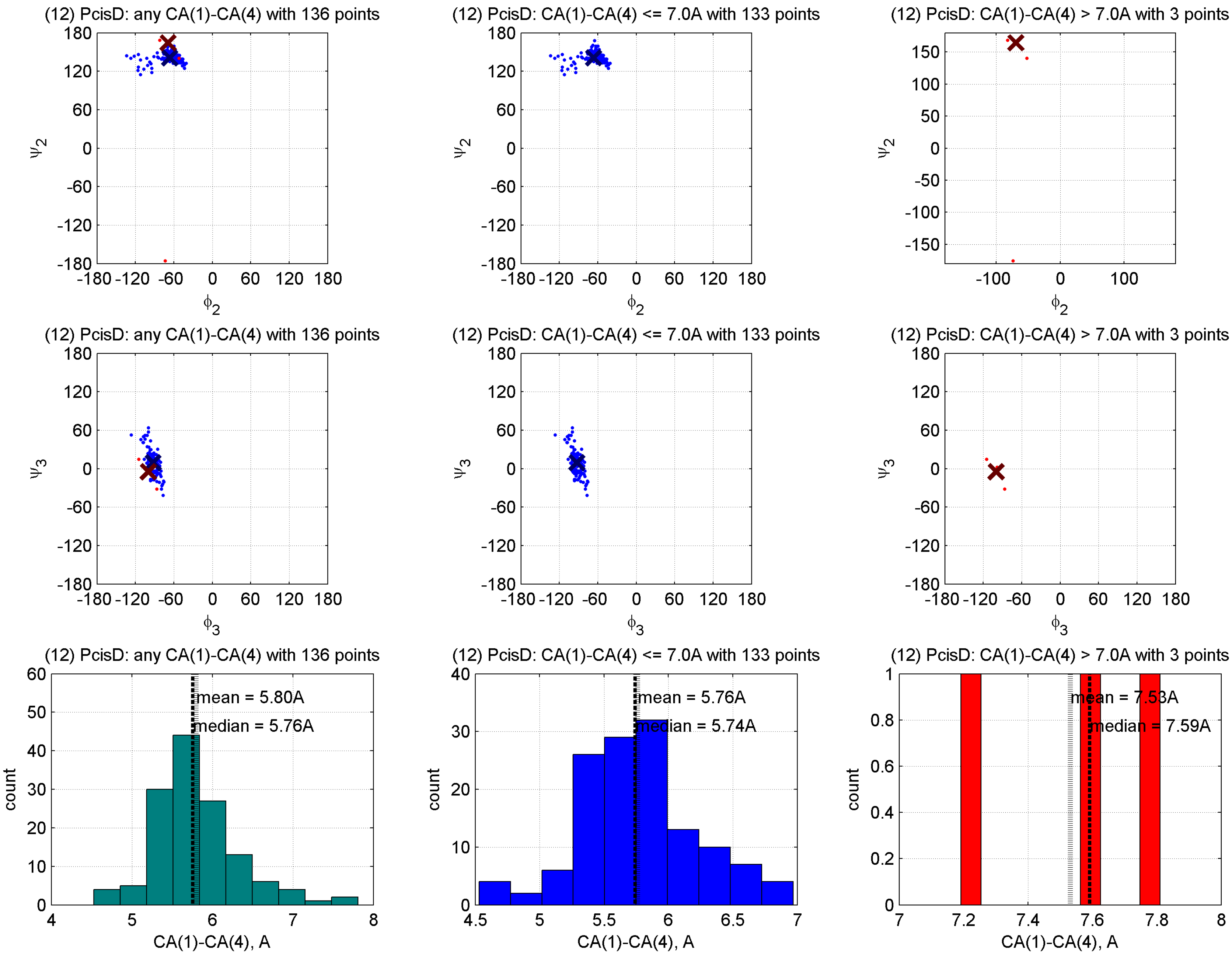
(12) PcisD: any CA(1)-CA(4) with 136 points
(12) PcisD: CA(1)-CA(4) <= 7.0A with 133 points
(12) PcisD: CA(1)-CA(4) > 7.0A with 3 points
ψ_2
ϕ_2
(12) PcisD: any CA(1)-CA(4) with 136 points
(12) PcisD: CA(1)-CA(4) <= 7.0A with 133 points
(12) PcisD: CA(1)-CA(4) > 7.0A with 3 points
ψ_3
ϕ_3
(12) PcisD: any CA(1)-CA(4) with 136 points
mean = 5.80A
median = 5.76A
(12) PcisD: CA(1)-CA(4) <= 7.0A with 133 points
mean = 5.76A
median = 5.74A
(12) PcisD: CA(1)-CA(4) > 7.0A with 3 points
mean = 7.53A
median = 7.59A
count
CA(1)-CA(4), A

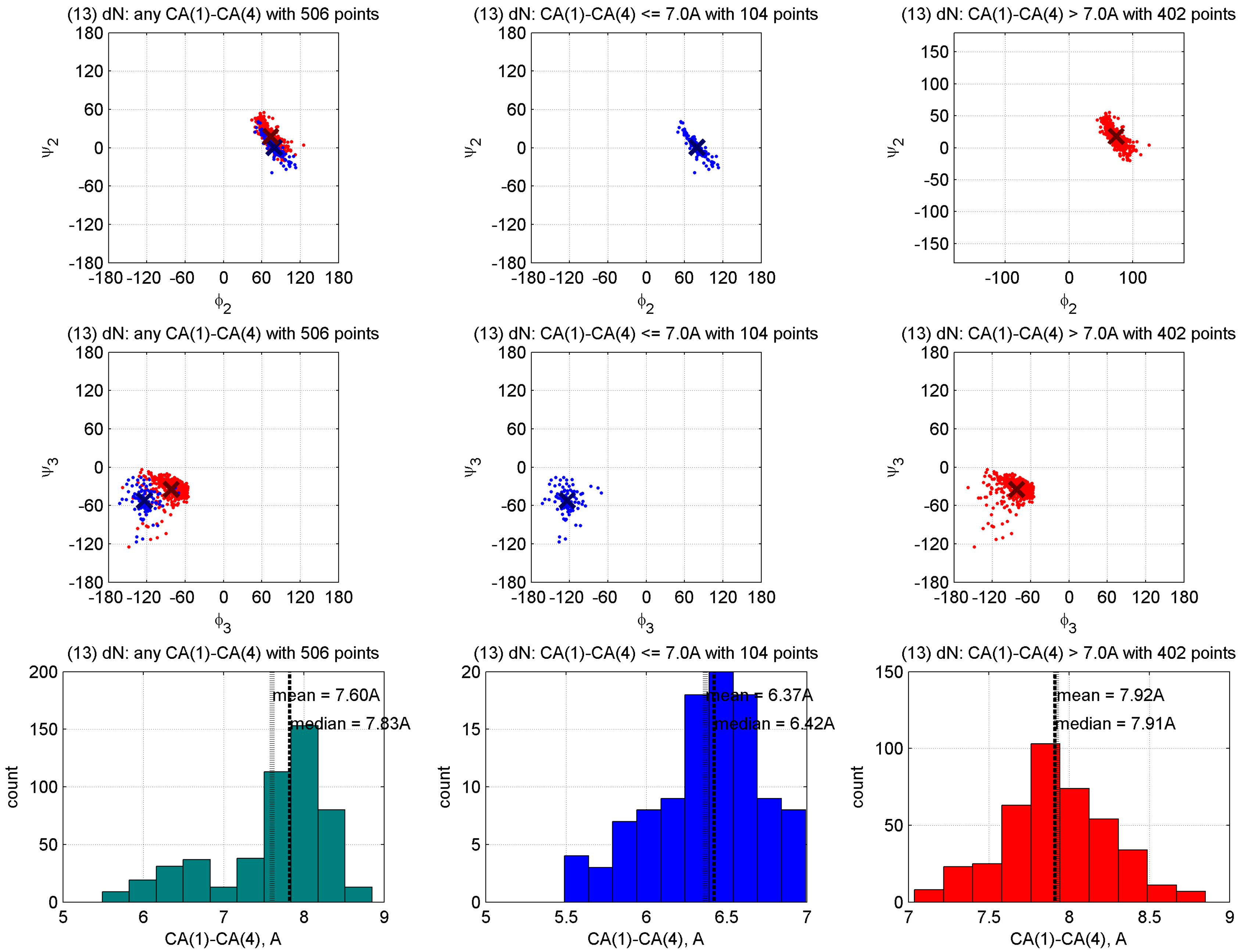

(13) dN: any CA(1)-CA(4) with 506 points
(13) dN: CA(1)-CA(4) <= 7.0A with 104 points
(13) dN: CA(1)-CA(4) > 7.0A with 402 points
ψ2
φ2
ψ3
φ3
count
CA(1)-CA(4), A
mean = 7.60A
median = 7.83A
mean = 6.37A
median = 6.42A
mean = 7.92A
median = 7.91A

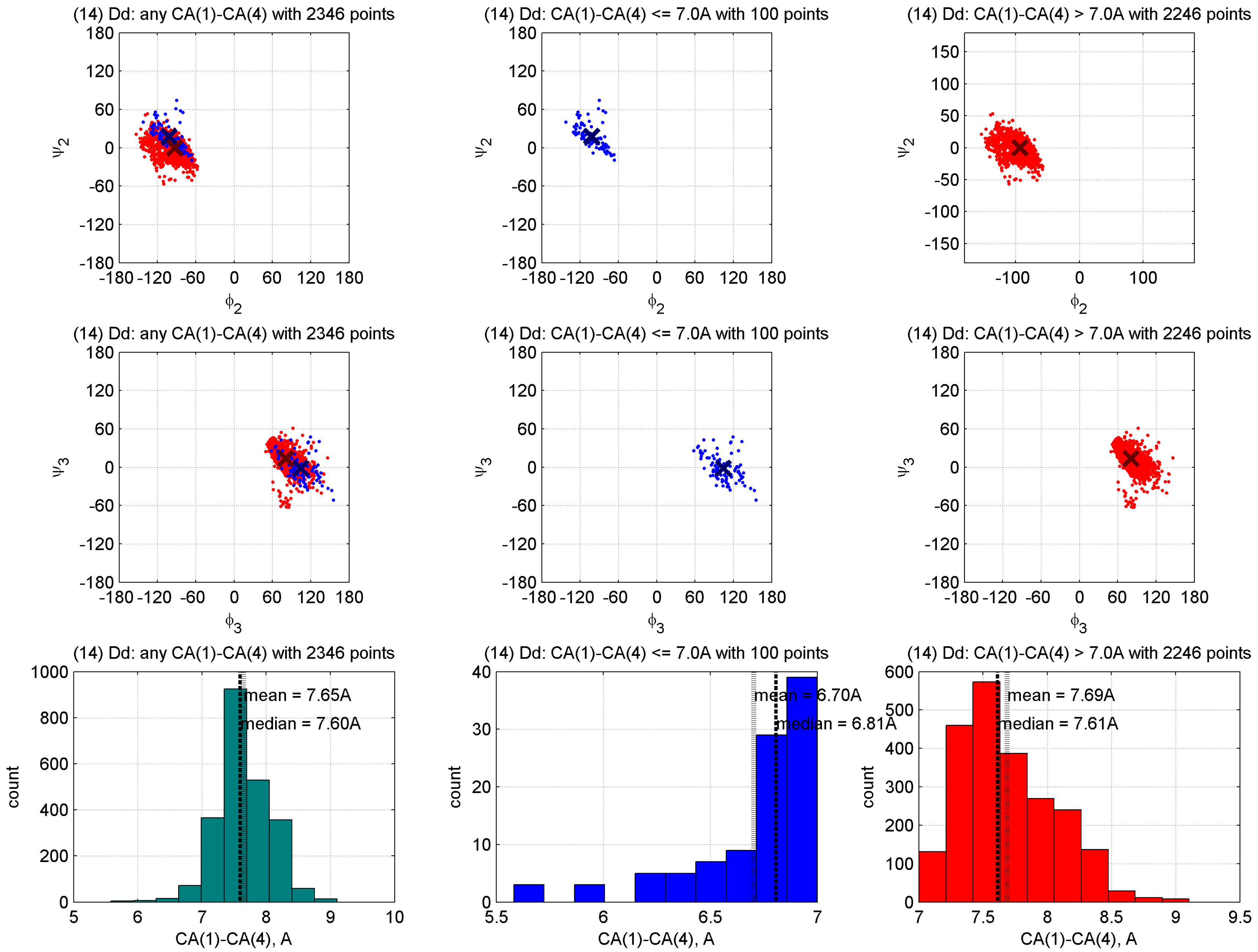

(14) Dd: any CA(1)-CA(4) with 2346 points
(14) Dd: CA(1)-CA(4) <= 7.0A with 100 points
(14) Dd: CA(1)-CA(4) > 7.0A with 2246 points
ψ2
ϕ2
ψ3
ϕ3
count
CA(1)-CA(4), A
mean = 7.65A
median = 7.60A
mean = 6.70A
median = 6.81A
mean = 7.69A
median = 7.61A

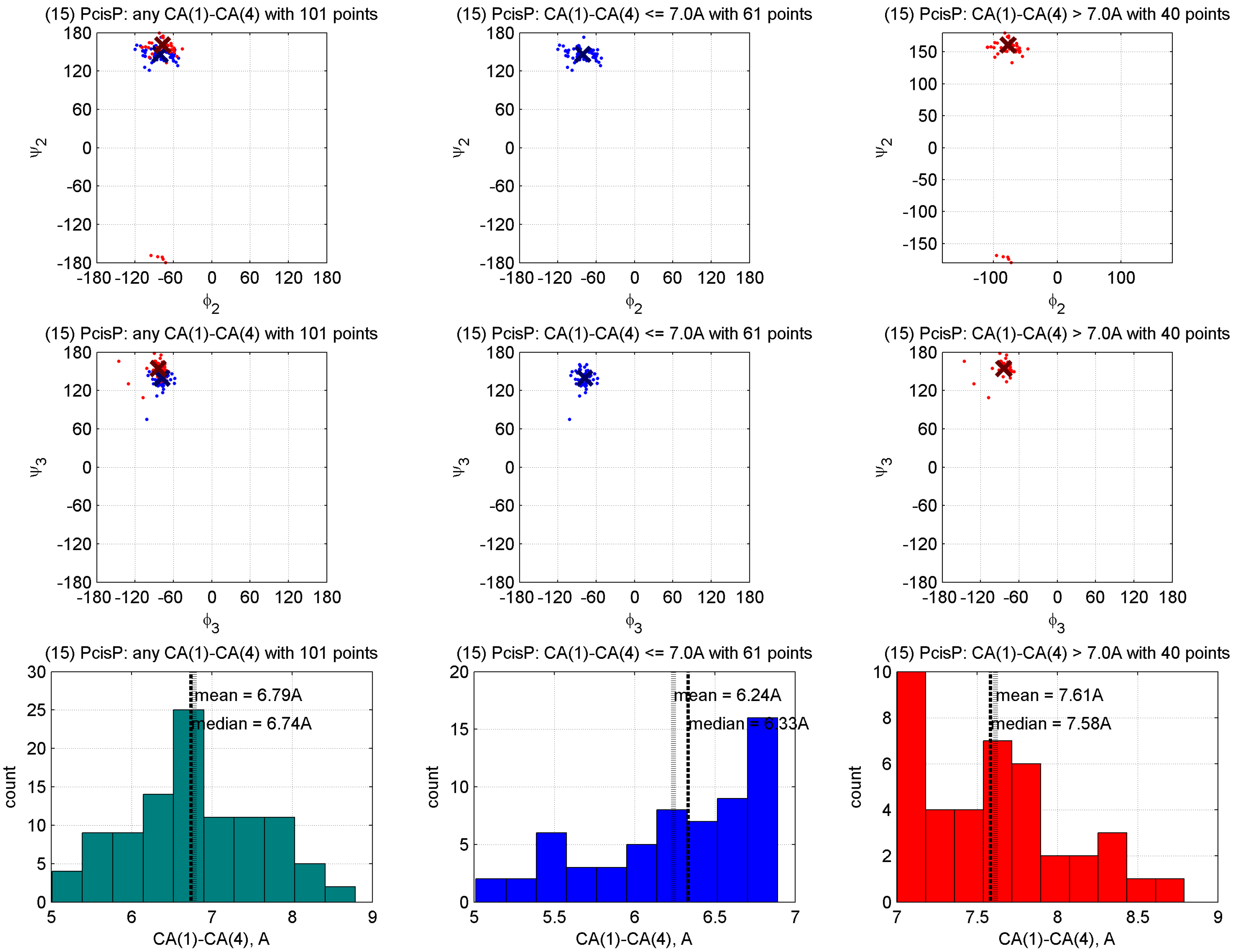
(15) PcisP: any CA(1)-CA(4) with 101 points
(15) PcisP: CA(1)-CA(4) <= 7.0A with 61 points
(15) PcisP: CA(1)-CA(4) > 7.0A with 40 points
ψ2
ϕ2
ψ3
ϕ3
count
CA(1)-CA(4), A
mean = 6.79A
median = 6.74A
mean = 6.24A
median = 6.33A
mean = 7.61A
median = 7.58A

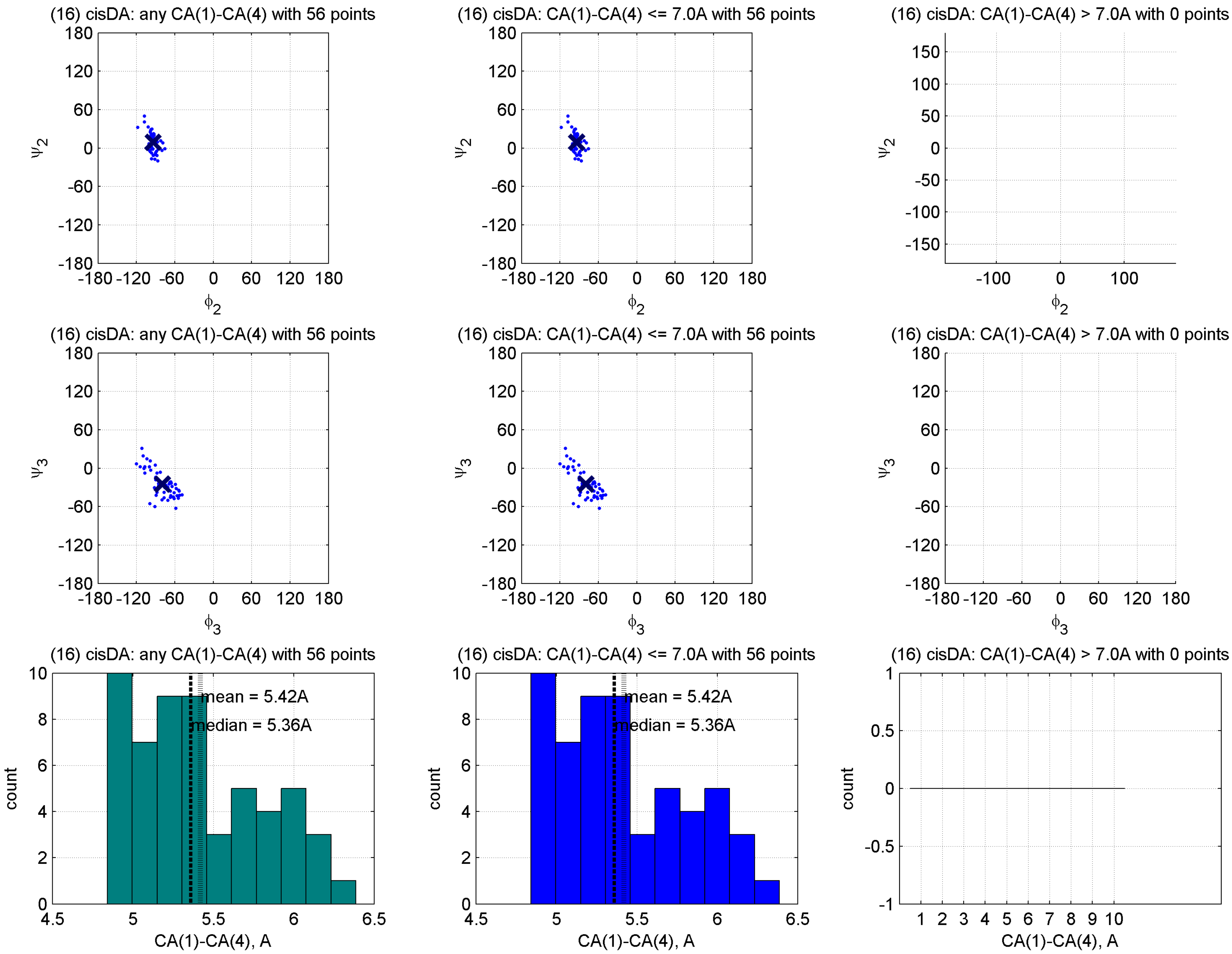

(16) cisDA: any CA(1)-CA(4) with 56 points
(16) cisDA: CA(1)-CA(4) <= 7.0A with 56 points
(16) cisDA: CA(1)-CA(4) > 7.0A with 0 points
ψ_2
ϕ_2
ψ_3
ϕ_3
count
CA(1)-CA(4), A
mean = 5.42A
median = 5.36A

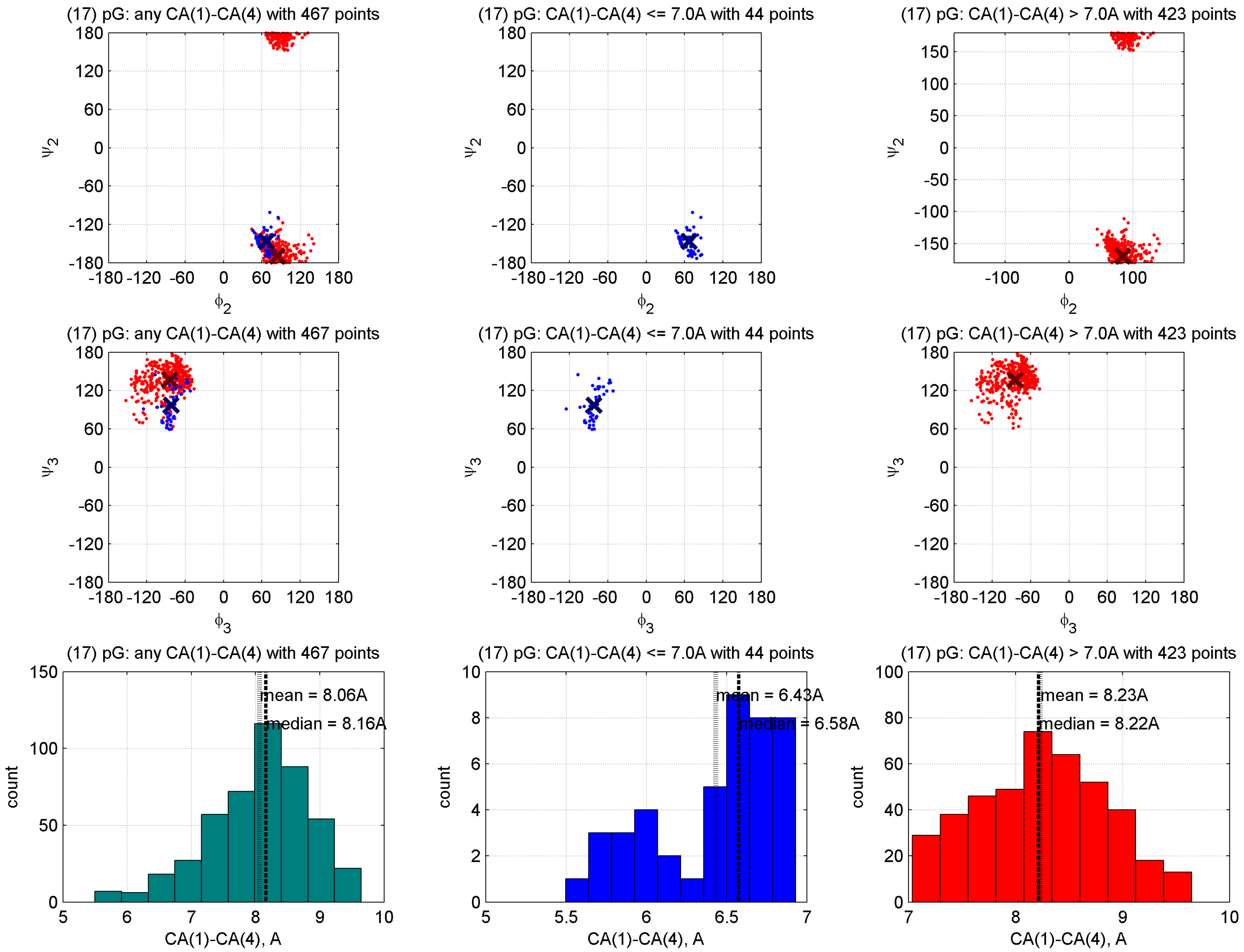
(17) pG: any CA(1)-CA(4) with 467 points
(17) pG: CA(1)-CA(4) <= 7.0A with 44 points
(17) pG: CA(1)-CA(4) > 7.0A with 423 points
ψ_2
ϕ_2
ψ_3
ϕ_3
mean = 8.06A
median = 8.16A
mean = 6.43A
median = 6.58A
mean = 8.23A
median = 8.22A
count
CA(1)-CA(4), A

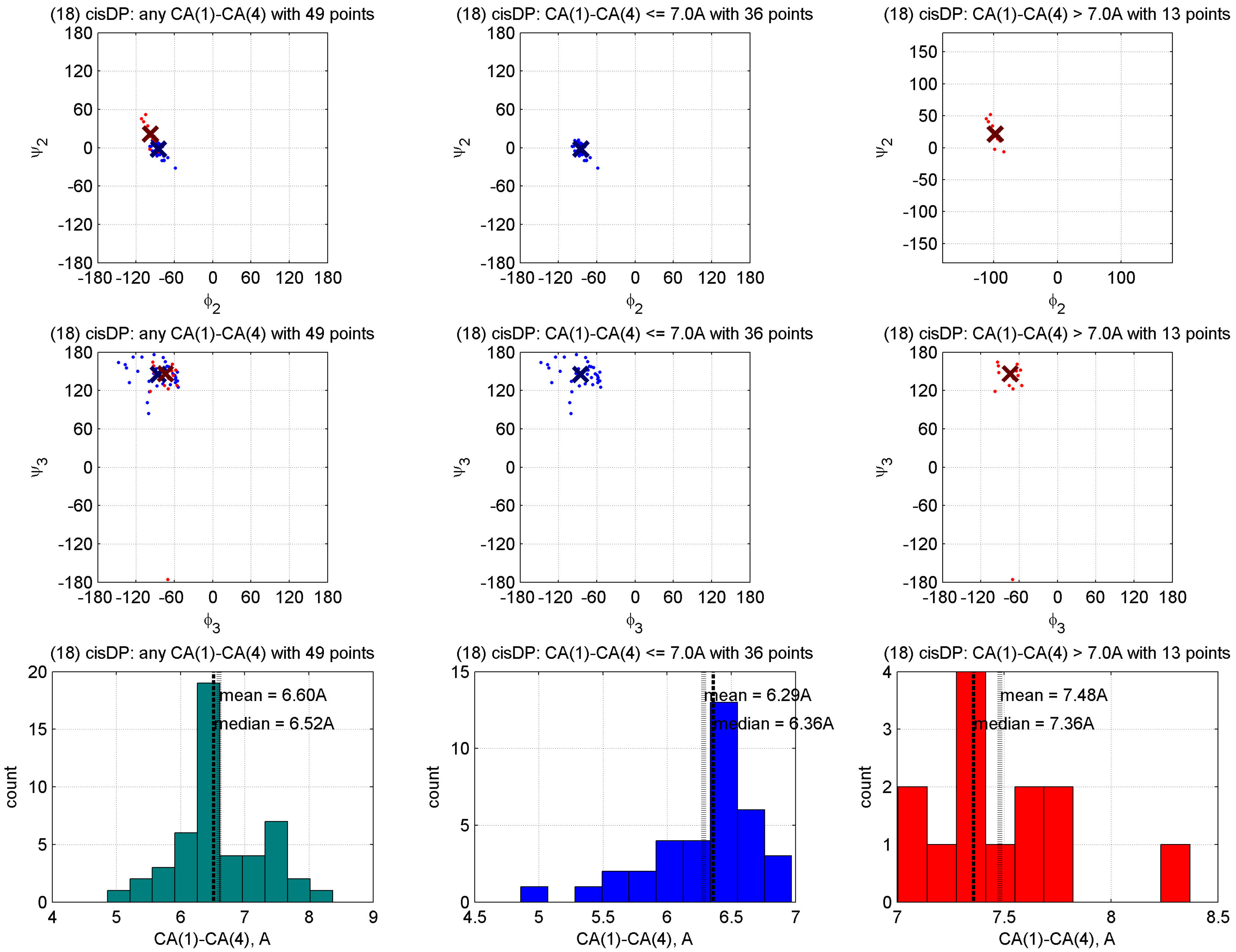

(18) cisDP: any CA(1)-CA(4) with 49 points
(18) cisDP: CA(1)-CA(4) <= 7.0A with 36 points
(18) cisDP: CA(1)-CA(4) > 7.0A with 13 points
ψ_2
ϕ_2
ψ_3
ϕ_3
count
CA(1)-CA(4), A
mean = 6.60A
median = 6.52A
mean = 6.29A
median = 6.36A
mean = 7.48A
median = 7.36A

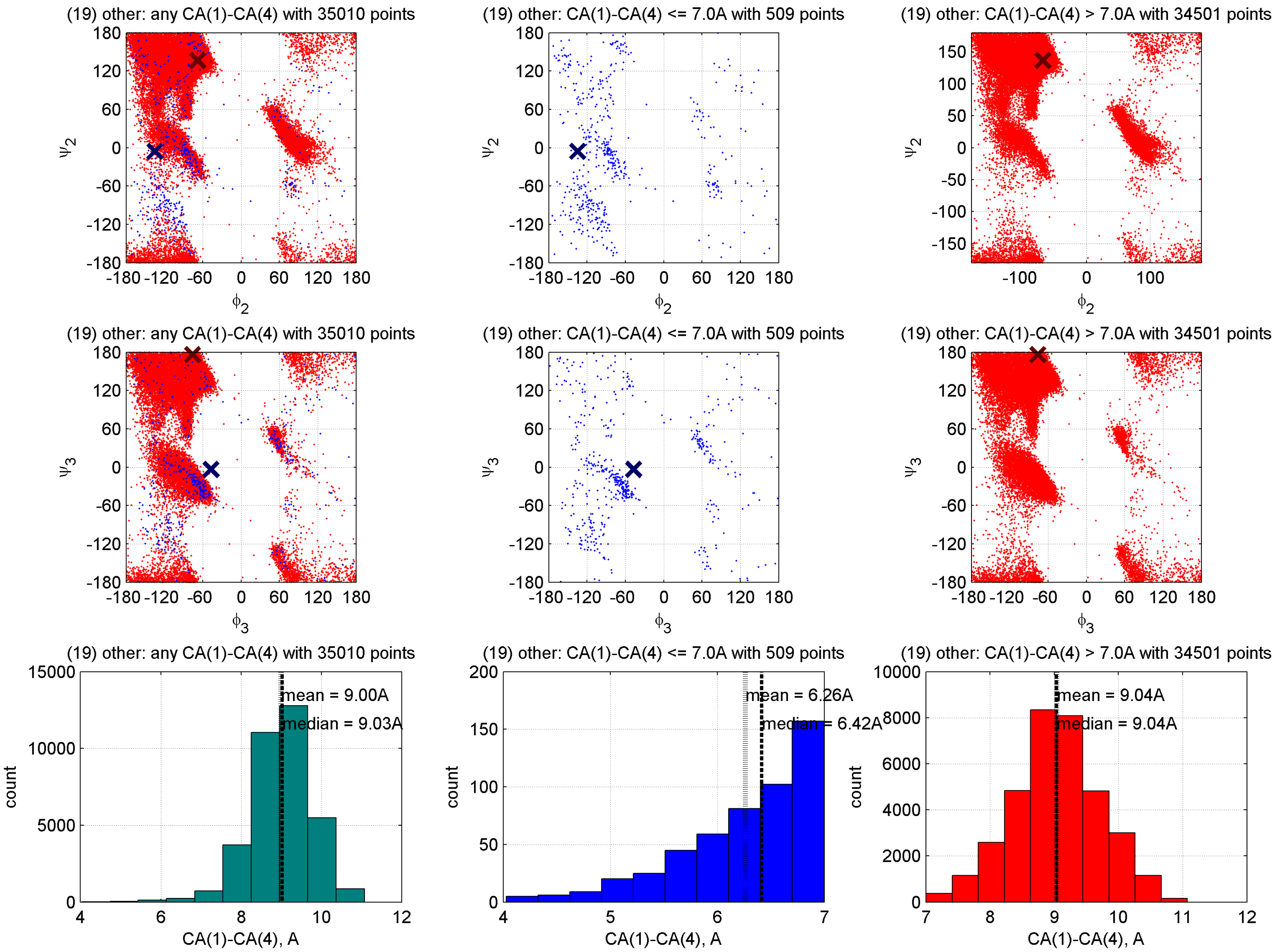
(19) other: any CA(1)-CA(4) with 35010 points
(19) other: CA(1)-CA(4) <= 7.0A with 509 points
(19) other: CA(1)-CA(4) > 7.0A with 34501 points
ψ_2
ϕ_2
ψ_3
ϕ_3
count
CA(1)-CA(4), A
mean = 9.00A
median = 9.03A
mean = 6.26A
median = 6.42A
mean = 9.04A
median = 9.04A
